# Adsorption of O_2_ on the Preferred -O-Au Sites of Small Gold Oxide Clusters: Charge-dependent Interaction and Activation

**DOI:** 10.3390/molecules29071645

**Published:** 2024-04-06

**Authors:** Lulu Huang, Wen Liu, Xiaopeng Xing

**Affiliations:** Shanghai Key Lab of Chemical Assessment and Sustainability, Department of Chemistry, Tongji University, 1239 Siping Road, Shanghai 200092, China; huanglulu@tongji.edu.cn (L.H.); 2410277@tongji.edu.cn (W.L.)

**Keywords:** gold oxide clusters, charge-dependence, oxygen activation, active sites

## Abstract

Decades of research have illuminated the significant roles of gold/gold oxide clusters in small molecule catalytic oxidation. However, many fundamental questions, such as the actual sites to adsorb and activate O_2_ and the impact of charge, remain unanswered. Here, we have utilized an improved genetic algorithm program coupled with the DFT method to systematically search for the structures of Au_1–5_O*_x_*^−/+/0^ (*x* = 1–4) and calculated binding interactions between Au_1–5_O*_x_*^−/+/0^ (*x* = 1–2) and O_2_, aiming to determine the active sites and to elucidate the impact of different charge states in gold oxide systems. The results revealed that the reactivity of all three kinds of small gold oxide clusters toward O_2_ is strongly site-dependent, with clusters featuring an -O-Au site exhibiting a preference for adsorption. The charges on small gold oxide clusters significantly impact the interaction strength and the activation degree of adsorbed O_2_: in the case of anionic cluster, the interaction between O_2_ and the -O-Au sites leads to a chemical reaction involving electron transfer, thereby significantly activating O_2_; in neutral and cationic clusters, the adsorption of O_2_ on their -O-Au sites can be viewed as an electrostatic interaction. Pointedly, for cationic clusters, the highly concentrated positive charge on the Au atom of the -O-Au sites can strongly adsorb but hardly activate the adsorbed O_2_. These results have certain reference points for understanding the gold oxide interfaces and the improved catalytic oxidation performance of gold-based systems in the presence of atomic oxygen species.

## 1. Introduction

Catalysis plays a substantial role in agricultural, industrial, and environmental fields, serving as one of the pivotal topics within the scope of chemical research. Notably, metallic nanocatalysts loaded on various oxides represent one of the most common forms of catalysts [1,2,3,4,5,6]. Gold, perceived as an exceedingly inert metal since ancient times, was discovered, by Haruta [7,8,9] and Hutchings [10,11] at the end of the last century, to exhibit remarkable catalytic oxidation activity toward CO and other small organic molecules. This groundbreaking discovery precipitated a surge in the amount of research related to gold systems, gradually culminating in the establishment of an independent field of study. 

Decades of research have illuminated the significant roles of gold nanoparticles, gold clusters, and even a single gold atom in small molecule catalytic oxidation [12,13,14,15,16,17]. However, many fundamental questions, such as the location of O_2_ adsorption and activation [12,18,19,20,21,22] and the impact of charge [3,20,23,24,25,26,27,28,29,30,31,32], still remain contested. Some studies suggest that the presence of gold at the oxide interface and gold oxides are key to O_2_ activation, and the introduction of these elements can improve the catalytic oxidation performance of these systems [32,33,34,35,36,37,38,39]. However, there is still a lack of detailed explanation at the atomic and molecular levels for specific sites and their mechanisms. In addition, researchers have found that gold clusters carrying either positive or negative charges both demonstrated catalytic activity in specific systems [23,26,27,28,29,30,31,32,33]. This suggests that various active sites with different charge states and spatial geometrical conditions might be involved in real catalytic processes [40]. In experiments, X-ray photoelectron spectroscopy is commonly utilized to assess charge transfer among nanomaterials. Nonetheless, conducting spectral analysis at such small scales poses a significant challenge and can even yield misleading results [3,41].

By adopting the cluster model which boasts advantages such as controllable preparation, simplicity, and close linkage with DFT theoretical computations, we can comprehend the mechanisms of heterogeneous catalysis interacting with small molecules at the atomic and molecular level [42,43,44,45]. Insights into the activation of O_2_ on gold-based catalysts have been derived from comprehensive studies on the reactions between various gold clusters and O_2_, merging data from numerous experiments and calculations [18,20,46,47,48,49,50,51,52,53,54,55]. Consistent findings indicate O_2_ functioning as a one-electron acceptor during its interactions with gold clusters, with electron transfer from gold to its anti-bonding π*_2p_ enhancing its adsorption and activation. The strong chemical interactions with O_2_ are primarily due to the unpaired electrons and the low electron binding energy inherent to gold clusters. Theoretical explorations have also been conducted on gold clusters containing one or two O atoms [53,55,56]. These O atoms tend to occupy terminal positions in clusters comprising no more than three gold atoms, or they bridge two peripheral gold atoms [56]. It has been generally observed that gold clusters featuring two separate O atoms maintain greater stability than their counterparts which adsorb an O_2_ molecule, particularly when the clusters incorporate more than three gold atoms [51,53,55]. Furthermore, some small gold oxide clusters, specifically AuO_1-2_^−^ and Au_2,4_O_2_^−^, have been generated within the plasma of the laser-vaporization cluster source. Combinatorial analysis using photoelectron spectrometry experiments and calculations determined their structures [52,57]. The interactions between Au*_x_*O*_y_*^+/−^ and CO have been thoroughly examined using a flow tube reactor, which illuminated several reaction channels [58,59,60,61,62]. We previously explored reactivity of AuO*_x_*^−^ (*x* = 1–3) with O_2_ and found that only AuO^−^ is active [63]. Recently, we have indicated that -O-Au is the preferred adsorption site for O_2_ on small anionic gold oxide clusters through theoretical calculations and mass spectrometry experiments, and the correlation with the global electronic characteristics is insignificant [64].

Compared with common cluster models, actual heterogeneous catalytic sites tend to be neutral or carry a small amount of charge due to charge transfer [30,65]. Therefore, revealing the relationships between cluster reactivity and the polarity and amount of charge on the clusters is crucial to scrutinize reaction mechanisms on actual catalysts based on cluster reaction results. This study aims to elucidate the impact of different charge states of gold oxide systems on O_2_ adsorption and activation, which is the rate-limiting step in many catalytic oxidation processes [32,66,67,68]. In this paper, we have carried out extensive theoretical calculations on the structures of Au_1–5_O_1,2_^−/+/0^ and the adsorption and activation of O_2_ on them. The results indicate that, no matter what the charge state of the cluster is, the -O-Au site is the preferred adsorption site for O_2_, with the largest binding energy. However, due to the different valence bond interactions formed on clusters’ various charge states, only the -O-Au sites in the anionic gold oxide clusters can significantly activate O_2_. 

## 2. Results and Discussion

### 2.1. Geometric Structures of Au_1–5_O_1,2_^−^ and Their Products with an O_2_

**Figure 1:**
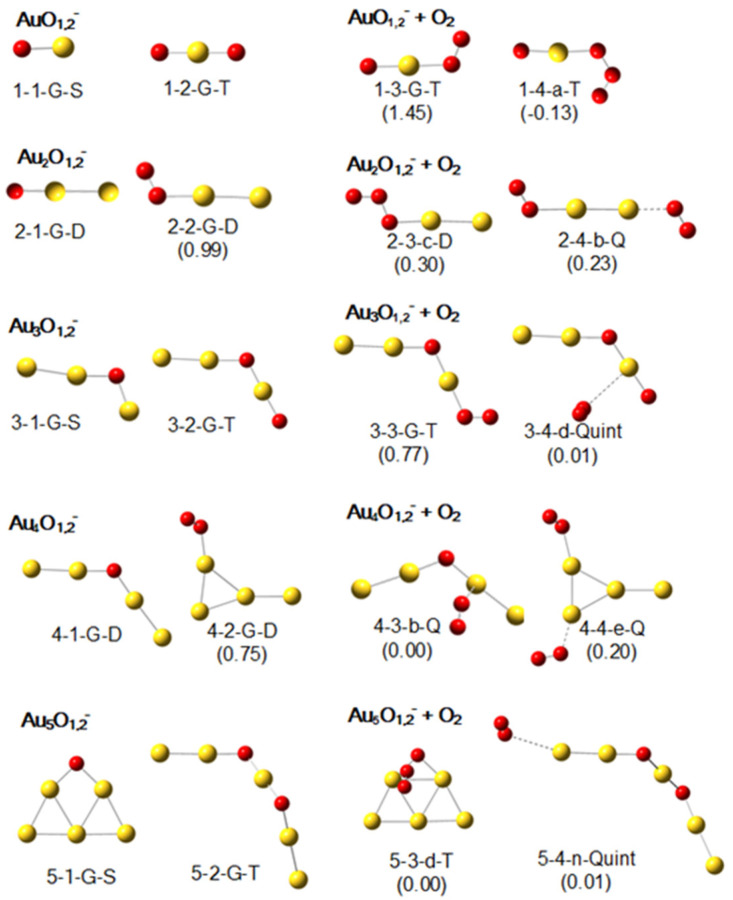
The lowest-lying structures of Au_1–5_O*_x_*^−^ (*x* = 1 and 2) and their most stable products after adsorbing one O_2_ according to calculations at the B3LYP level with the basis sets of def2-SVP for Au, and def2-TZVP for O. In the labels of the structures, the first two numerals indicate the number of gold atoms and the number of oxygen atoms, respectively; the third part “G/a/b…” means that this structure is the lowest-lying, the second lowest-lying, or the third lowest-lying one among all structural candidates; the fourth part indicates the spin-multiplicity, in which “S”, “D”, “T”, “Q”, and “Quint” stand for singlet, doublet, triplet, quartet, and quintet, respectively. The numerals in the parentheses following the labels of the structures containing adsorbed O_2_ unit(s) show the adsorption energies (E*_a_*, in eV) of the second O_2_.

The low-lying structures of Au_1–5_O_1,2_^−^ have been reported in our previous work [64], and Figure 1 shows the lowest-lying ones. For references to other low-lying structures, please refer to Figures S1–S4 in the Supplementary Materials. In Figure 1, the O atoms in Au_1–5_O^−^ are coordinated to one or two Au atoms, which is consistent with previous results [56]; for Au_1–5_O_2_^−^, when the number of Au atoms is odd, the energy of the oxide formation is energetically favorable (with O atoms dissociated), whereas when the number of Au atoms is even, the formation with an absorbed O_2_ becomes the most energetically favorable structure. The most energetically advantageous adsorption site for the O_2_ in Au_2,4_O_2_^−^ is consistent with previous theoretical results [49,50,51,55,69,70].

For the structures of Au_1–5_O_1,2_^−^, illustrated in the left two columns of Figure 1, only 1-1-G-S and 3-1-G-S have an -O-Au site, and adsorption of O_2_ on these two structures forms 1-3-G-T and 3-3-G-T with the largest two adsorption energies of 1.45 eV and 0.77 eV, respectively (in the right two columns of Figure 1). Similar situations can also be repeatedly confirmed in the Supplementary Materials, such as the O_2_ adsorption products of 1-4-G-T (E*_a_*: 1.05 eV), 2-3-b-D (E*_a_*: 1.35 eV), and 2-4-G-D (E*_a_*: 1.05 eV) in Figure S1. Also, for clusters with a slightly larger number of gold atoms in Figures S2–S4, we can find 3-4-b-T (E*_a_*: 1.15 eV), 3-4-c-T (E*_a_*: 0.87 eV), and 5-4-g-T (E*_a_*: 0.89 eV) and many other structures that comply with the adsorption rules. Namely, if the -O-Au site is present, the adsorption energy of O_2_ on it is the largest (approx. 0.5–1.5 eV). However, if there is no such site, the adsorption of O_2_ is extremely weak, as demonstrated by 3-4-d-Quint (E*_a_*: 0.01 eV), 4-3-b-Q (E*_a_*: 0.00 eV), and 5-4-n-Quint (E*_a_*: 0.01 eV) in Figure 1. Additional examples for this weak interaction can be found in the Supplementary Materials (Figures S1–S4).

### 2.2. Geometric Structures of Au_1–5_O_1,2_^+^ and Their Products with an O_2_

**Figure 2:**
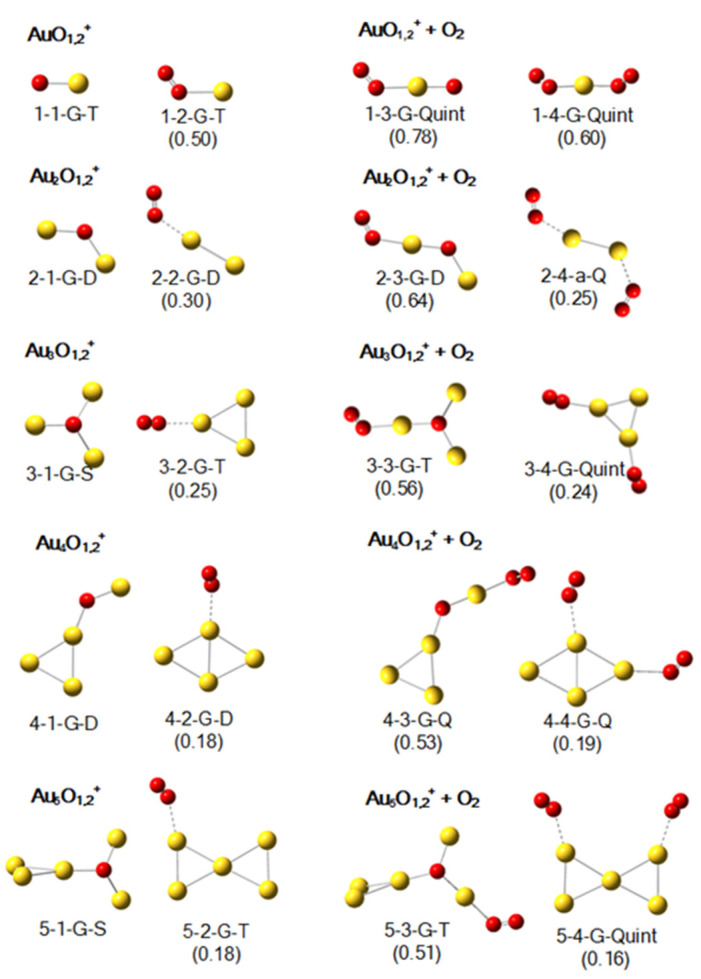
The lowest-lying structures of Au_1–5_O*_x_*^+^ (*x* = 1 and 2) and their most stable products after adsorbing one O_2_ according to calculations at the B3LYP level with the basis sets of def2-SVP for Au, and def2-TZVP for O. The meanings of the labels and the numerals are the same as those in Figure 1.

The lowest-lying structures of Au_1–5_O_1,2_^+^ are shown in the left two columns of Figure 2. In the geometric structures of Au_2–5_O^+^, the O atom connects three gold atoms when the number of Au atoms is odd, and it connects two gold atoms when the number of Au atoms is even. Our computational work remains consistent with previous work [56]. For Au_1–5_O_2_^+^, the lowest-lying structures can be interpreted as the lowest-lying cationic pure gold cluster adsorbed an O_2_ [71,72,73]. The adsorption energies of O_2_ clearly show that, with the exception of AuO_2_^+^ (E*_a_*: 0.50 eV), the adsorption interaction here is relatively low, which is consistent with the results of Ding et al. [70]. Additionally, there is a decreasing trend in adsorption energies as the number of gold atoms increases, which may be related to what we will discuss later: for cationic gold oxide clusters and neutral gold oxide clusters, the adsorption energy of O_2_ on them is proportional to the amount of positive charge on the Au atom of the -O-Au sites.

As shown in the right two columns of Figure 2, the adsorption energies of O_2_ on the -O-Au sites of 1-1-G-T and 2-1-G-D remain high, at approximately 0.78 eV (1-3-G-Quint) and 0.64 eV (2-3-G-D), respectively. The adsorption energies of O_2_ on the -O-Au sites of 3-1-G-S, 4-1-G-D and 5-1-G-S are 0.56 eV (3-3-G-T), 0.53 eV (4-3-G-Q), and 0.51 eV (5-3-G-T), respectively. This observed preference is similar to our prior findings regarding anionic gold oxide clusters [64]. Apart from the ones shown in Figure 2, Figures S5–S8 in the Supplementary Materials provide other examples to repeatedly confirm this preference (such as the adsorbed O_2_ in 2-4-G-Q, 2-4-c-D, 3-3-b-Quint, 3-4-a-Quint and 5-4-a-Quint). In a word, the adsorption energy of O_2_ on the -O-Au site is higher than that on other sites, with the majority of clusters’ adsorption energies around 0.5 eV.

In the absence of the -O-Au site, the adsorption will be weak. As portrayed in Figure 2, the structures 2-4-a-Q, 3-4-G-Quint, 4-4-G-Q, and 5-4-G-Quint exhibit adsorption energies of merely 0.25 eV, 0.24 eV, 0.19 eV, and 0.16 eV, respectively. In Figure 2, the Au_1–5_O_4_^+^ clusters can be regarded as pure gold clusters adsorbing two molecular oxygen. The values of adsorption energies in this study align well with those reported by Ding et al., with only minor discrepancies observed in the adsorption sites of 4-4-G-Q and 5-4-G-Quint [70]. As shown in Figure S6, the 3-2-b-T encompasses both an -O-Au site and an -O-Au-Au site, and the adsorption energy at the active site is 0.51 eV (3-4-b-Quint), whereas that on the -O-Au-Au site is only 0.10 eV (3-4-c-Quint). In Figure S7, 4-1-b-D has a gold triangle and an -O-Au site, and the adsorption energy of O_2_ on its gold triangle and -O-Au site is calculated to be 0.17 eV (4-3-g-D) and 0.50 eV (4-3-d-Q), respectively. An analogous weak adsorption scenario can be observed in many other adsorption structures like 3-3-g-Quint, 5-3-a-T, and 5-4-c-Quint in Figures S6 and S8.

### 2.3. Geometric Structures of Au_1–5_O_1,2_^0^ and Their Products with an O_2_

**Figure 3:**
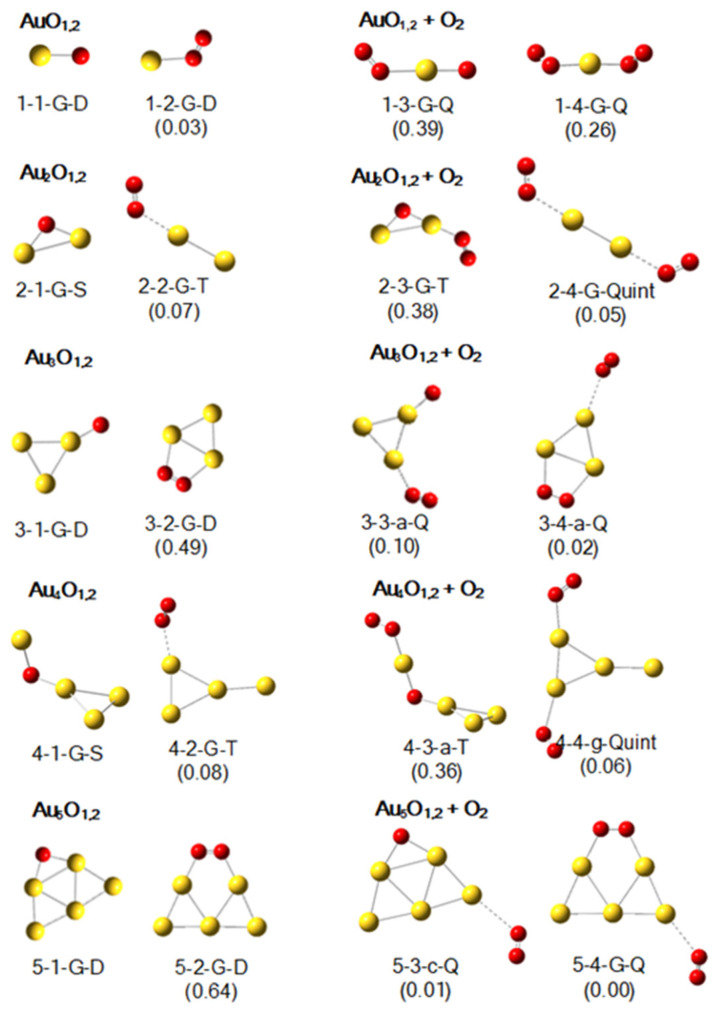
The lowest-lying structures of Au_1–5_O*_x_* (*x* = 1 and 2) and their most stable products after adsorbing one O_2_ according to calculations at the B3LYP level with the basis sets of def2-SVP for Au, and def2-TZVP for O. The meaning of the labels and the numerals are same as those in Figure 1.

The lowest-lying structures of Au_1–5_O_1,2_ are shown in the left two columns of Figure 3. For the clusters with one or three gold atoms, the O is mono-coordinated, whereas when the number of gold atoms is equal to 2, 4, or 5, the O atom is di-coordinated. The lowest-lying structures obtained by our calculations are consistent with previously reported results [56]. For Au_1–5_O_2_, the lowest-lying structures are those with molecular oxygen adsorbed onto pure gold clusters. It is noteworthy that when the number of gold atoms is three or five, peroxide adsorption forms, with adsorption energies of 0.49 eV and 0.64 eV being found. These two peroxide structures are consistent with previous results [70], and their O_2_ units are highly activated: the O-O bond length, O-O vibration frequency, and NPA charge on O_2_ are 1.281 Å, 1199.00 cm^−1^, and −0.427 a.u. for Au_3_O_2_, and 1.316 Å, 1161.80 cm^−1^, and −0.540 a. u. for Au_5_O_2_.

As shown in the right two columns of Figure 3, the corresponding adsorption structures of 1-1-G-D, 2-1-G-S, and 4-1-G-S are 1-3-G-Q, 2-3-G-T, and 4-3-a-T, respectively. The original structures all have active -O-Au sites, and the adsorption energies of O_2_ on these sites are 0.39 eV, 0.38 eV, and 0.36 eV, respectively. Aside from those shown in Figure 3, there are other examples in the Supplementary Materials. As can be seen in Figure S9, the corresponding adsorption structure of 2-2-a-T is 2-4-b-Quint, and the adsorption energy on its -O-Au site is 0.40 eV. Some additional examples include 3-4-b-Q and 3-4-e-Q from Figure S10; 4-3-e-T, 4-3-h-T and 4-4-j-Quint from Figure S11; and 5-4-i-Q from Figure S12. In a word, if there is an -O-Au site, the adsorption energy of O_2_ is maximal (relative to other sites), and the adsorption energies on these sites are typically slightly less than 0.4 eV.

If there is no -O-Au site, adsorption of O_2_ is extremely weak. As depicted in Figure 3, the corresponding adsorption structures of 3-1-G-D and 5-1-G-D are 3-3-a-Q and 5-3-c-Q with the adsorption energies of 0.10 eV and 0.01 eV, respectively. There are other examples in the Supplementary Materials (Figures S9–S12), which are not all enumerated. The adsorption energies of the second O_2_ on Au_1-5_O_2_ are nearly identical to the previous theoretical results predicted by Ding et al. [70].

### 2.4. Charge-Dependent Bonding Strengths and Activation Degrees

To summarize and compare the adsorption energies and the activation degree of O_2_ on the -O-Au sites in the structures depicted in Figure 1, Figure 2 and Figure 3, we present related calculated parameters of the adsorbed O_2_ in Table 1. Anionic gold oxide clusters exhibit the largest binding energies for O_2_ among the three series, along with the longest O-O bond lengths (above 1.32 Å). The calculated bond length of a free O_2_ stands at 1.204 Å (1.208 Å as reported in an experiment by [74]), so anionic gold oxide clusters show a significant stretching of the O-O bond. Simultaneously, the O_2_ units on anionic gold oxide clusters accumulate more than 0.6 a.u. negative charges, and their spins are close to 1.0. All these parameters indicate that the adsorbed O_2_ on the -O-Au sites of these anionic gold oxide clusters gain an electron onto its π* anti-bonding orbital, which significantly activates the O-O bond. For cationic gold oxide clusters, apart from 1-3-G-Quint and 2-3-G-D, which have adsorption energies of 0.78 eV and 0.64 eV, respectively, the rest of the structures tend to have adsorption energies slightly higher than 0.50 eV. The O-O bond lengths of the adsorbed O_2_ on these cationic gold oxide clusters are around 1.21 Å, which is very close to that of a free O_2_; the adsorbed O_2_ units are slightly positively charged, and their spins are close to that of free O_2_. All these parameters indicate that the O_2_ units on the -O-Au sites of the cationic gold oxide clusters are almost not activated. Neutral gold oxide clusters show adsorption energies slightly lower than 0.4 eV, with corresponding O-O bond lengths distributed around 1.225 Å, which is between those of the aforementioned anionic and cationic ones. Their O_2_ units carry a slight negative charge. Compared to free O_2_, the spins of the adsorbed O_2_ on these neutral gold oxide clusters decrease a little, implying the weak activation of O_2_ on the neutral species despite its relatively weak binding.

**Figure 4:**
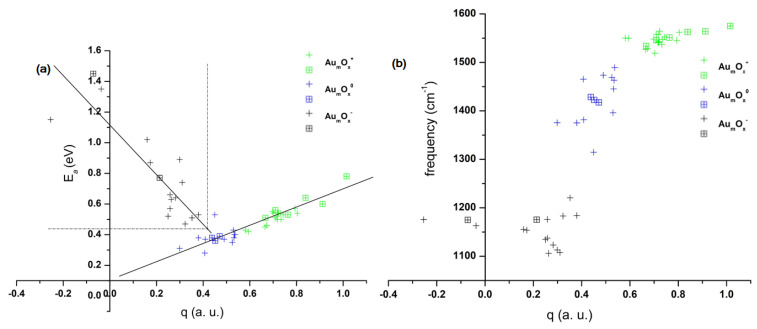
(**a**) The correlations between the adsorption energies (E*_a_*) of O_2_ and the NPA charges localized on the Au atoms of -O-Au sites and (**b**) the correlations between the frequencies (cm^−1^) of the adsorbed O_2_ and the NPA charges localized on the Au atoms of the -O-Au sites. The data are those of the lowest-lying structures of Au_1–5_O*_x_*^−/+/0^ (*x* = 1 and 2) shown in Figure 1, Figure 2 and Figure 3 (the square symbols 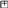) and other structures (the cross symbols +) with the -O-Au sites in the Supplementary Materials. The results of the anionic, the neutral, and the cationic species are shown by the black, the blue, and the green symbols, respectively.

In Figure 4a,b, we summarized the variations of the adsorption energies (E*_a_*) and the stretching frequencies of the adsorbed O_2_ vs. the NPA charges localized on the Au atom of the -O-Au sites in Au_1–5_O*_x_*^−/+/0^ (*x* = 1 and 2). The considered structures include the lowest-lying ones shown in Figure 1, Figure 2 and Figure 3 as well as other examples shown in Figures S1–S12. For anionic Au_1–5_O*_x_*^−^ (*x* = 1 and 2), a roughly inverse correlation was observed between the adsorption energies (E*_a_*) and the NPA charges. The E*_a_* values decrease from around 1.5 eV to around 0.5 eV when the NPA charges increase from around −0.1 a.u. to around +0.4 a.u. The stretching frequencies of the adsorbed O_2_ on the -O-Au sites concentrate in the range of 1100 to 1200 cm^−1^. These values are much lower than the calculated stretching frequencies of a free O_2_, which stands at 1637 cm^−1^ (1580 cm^−1^ as reported in experiment [74]), and there is not a clear correlation between these frequencies and the NPA charges. For cationic gold oxide clusters Au_1–5_O*_x_*^+^ (*x* = 1 and 2), an approximately positive correlation exists between the E*_a_* values and the NPA charges on the Au atoms of -O-Au sites. The E*_a_* values increase from around 0.4 eV to around 0.8 eV when the NPA charges increase from around +0.6 a.u. to around +1.0 a.u. The stretching frequencies of the adsorbed O_2_ on the -O-Au sites concentrate in the range of 1500 to 1600 cm^−1^. These values are very close to that of a free O_2_, and there is not a clear correlation between these frequencies and the NPA charges. For neutral Au_1–5_O*_x_* (*x* = 1 and 2), the adsorption energies (E*_a_*) concentrate around 0.4 eV, which is lower than the E*_a_* values of the anionic and cationic Au_1–5_O*_x_*^−/+^ (*x* = 1 and 2). The correlation between E*_a_* and NPA charges of neutral Au_1–5_O*_x_* (*x* = 1 and 2) can be viewed as an extension of the positive correlation of cationic Au_1–5_O*_x_*^+^ (*x* = 1 and 2) toward the small NPA charge values. The stretching frequencies of the adsorbed O_2_ on the -O-Au sites in neutral clusters spread from 1300 cm^−1^ to 1500 cm^−1^, which is between the values of the anionic and cationic species.

### 2.5. Analyses on the Bonding Patterns

**Figure 5:**
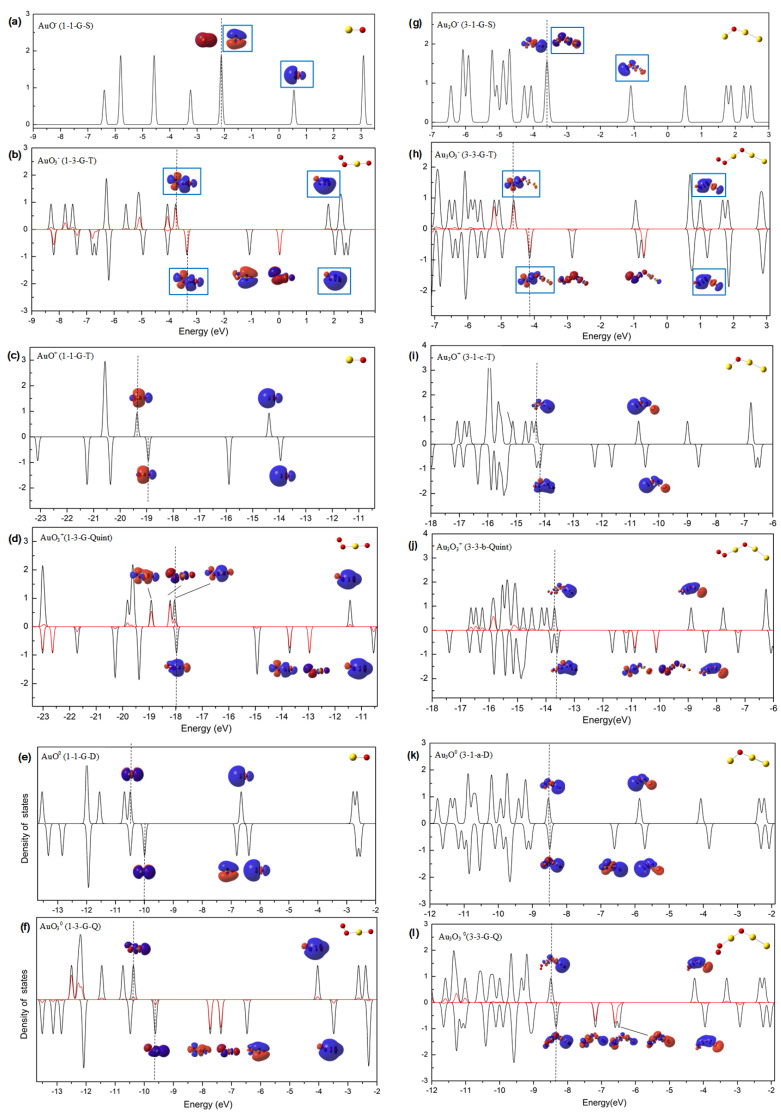
The DOS and PDOS (O_2_) figures in (**a**) AuO^−^ (1-1-G-S) and (**b**) AuO_3_^−^ (1-3-G-T); (**c**) AuO^+^ (1-1-G-T) and (**d**) AuO_3_^+^ (1-3-G-Quint); (**e**) AuO (1-1-G-D) and (**f**) AuO_3_ (1-3-G-Q); (**g**) Au_3_O^−^ (3-1-G-S) and (**h**) Au_3_O_3_^−^ (3-3-G-T); (**i**) Au_3_O^+^ (3-1-c-T) and (**j**) Au_3_O_3_^+^ (3-3-b-Quint); (**k**) Au_3_O (3-1-a-D) and (**l**) Au_3_O_3_ (3-3-G-Q). In each panel, the DOSs and PDOSs (O_2_) are plotted in black and in red, respectively; the HOMO position is indicated by a dotted line. The figures of some orbitals (those around the HOMO position) are shown to illustrate the bonding interactions between the gold oxide clusters and O_2_, and the orbitals enclosed by blue frames stand for the ones most correlated with these interactions. These results were obtained according to the KS orbitals from the calculations at the B3LYP level with the basis sets of def2-SVP for Au and def2-TZVP for O.

In order to understand the charge-dependent bonding strengths and activation degrees of O_2_ on the -O-Au sites of various clusters, we conducted an analysis of the density of states (DOSs). This analysis allowed us to identify the bonding patterns in several representative structures with differing charge polarities. The density of states (DOSs) of AuO^−^ (1-1-G-S), AuO^+^ (1-1-G-T), AuO (1-1-G-D), Au_3_O^−^ (3-1-G-S), Au_3_O^+^ (3-1-c-T), and Au_3_O (3-1-a-D) are shown in Figure 5a,c,e,g,i,k, respectively. The density of states (DOSs) of their adsorption products, AuO_3_^−^ (1-3-G-T), AuO_3_^+^ (1-3-G-Quint), AuO_3_ (1-3-G-Q), Au_3_O_3_^−^ (3-3-G-T), Au_3_O_3_^+^ (3-3-b-Quint), and Au_3_O_3_ (3-3-G-Q), and the partial density of states (PDOSs) of the adsorbed O_2_ are shown in Figure 5b,d,f,h,j,l, respectively.

Insights from the results of AuO^−^ (1-1-G-S), AuO_3_^−^ (1-3-G-T), Au_3_O^−^ (3-1-G-S), and Au_3_O_3_^−^ (3-3-G-T) presented in Figure 5a,b,g,h, reveal that the two up-spin and one down-spin components originating from the π_2p_* of O_2_ are occupied in the adsorption products. These observations suggest that a single electron has been transferred from the anionic gold oxide clusters to the adsorbed O_2_, which follows a pattern reminiscent of O_2_ adsorption on active Au*_n_*^−^ [51]. It is crucial to note that an excess electron on the π_2p_* of O_2_ may substantially weaken the O-O bond strength, echoing the findings shown in Table 1 and Figure 4. The interaction process between AuO^−^ (1-1-G-S) or Au_3_O^−^ (3-1-G-S) and O_2_ can be elaborated as follows: an electron located on one HOMO (π*_//_) of the anionic cluster is excited to its LUMO (the σ orbital enclosed by a blue frame). Consequently, the occupied σ orbital, which extends externally, showcases a high propensity for σ bond formation. Subsequently, the interaction between this σ orbital and one singly occupied π* orbital of O_2_, results in an occupied σ orbital and an unoccupied σ orbital. This newly formed occupied σ orbital boasts bonding characters predominantly comprised of the π* orbital of O_2_, and the newly formed unoccupied σ orbital exhibits antibonding characters mainly originating from the LUMO of AuO^−^ or Au_3_O^−^. These chemical bonding figures can account for the strong bonding strengths and the high activation degrees of O_2_ in these anionic species. 

According to the calculated DOSs of AuO^+^ (1-1-G-T), AuO_3_^+^ (1-3-G-Quint), Au_3_O^−^ (3-1-c-T), and Au_3_O_3_^+^ (3-3-b-Quint) shown in Figure 5c,d,i,j, the up-spin and down-spin components on the cluster moiety and the O_2_ do not apparently change during the adsorption reactions, and marginal electron transfer can be distinguished. The bonding interaction between the two moieties could be related to mixing of the filled up-spin components of gold oxide and the filled up-spin π* components of O_2_. For the corresponding neutral examples, AuO (1-1-G-D), AuO_3_ (1-3-G-Q), Au_3_O (3-1-a-D), and Au_3_O_3_ (3-3-G-Q), their calculated DOSs shown in Figure 5e,f,k,l, present scenarios similar to those of cations. The main contributions to the bonding interactions in these cationic and neutral species can be attributed to electrostatic attractions between the more or less positively charged Au atom of -O-Au sites and the polarized O_2_ molecule. The more positively charged Au atom of -O-Au sites in the cationic gold oxide clusters can interpret their marginally stronger bonding strengths with O_2_ than those in the neutral ones. At the same time, the more positively charged Au atom of -O-Au sites can more effectively prevent electron transfer to the π* orbital of O_2_ and therefore lead to the lower activation degrees of O_2_ in the cationic ones. 

## 3. Methods

The structures of Au_1–5_O*_x_*^−/+/0^ (*x* = 1–4) were preliminarily identified using a modified version of the Deaven–Ho genetic algorithm [35,75,76,77]. The modification involved incorporating incomplete optimizations of descendant structures from each crossover and mutation step [78]. The reliability, feasibility, and efficiency of this algorithmic procedure have been demonstrated in our previous published articles [64,78,79].

The specific implementation process is as follows:

(1) In our search program, we specify the number of gold and oxygen atoms and the multiplicity of the clusters. Based on the complexity of cluster searching, we determine the type and number of initial structures as initial random structures with diverse motifs. We have designed a module capable of generating seven typical motifs for a defined cluster size: the space-free motif, the close packing motif, the simple cubic packing motif, the cage motif, the solid sphere motif, the ring motif, and the specially defined motif through atomic coordinates. The latter allows users to input specially defined or previously reported structures.
(2) The initial random structures undergo relaxation using an incomplete optimization approach and are screened using the competition method under the small basis set we specify. The surviving structures become the offspring of the first generation.
(3) The first-generation results undergo multiple iterations of crossover and mutation under the genetic algorithm framework, generating a substantial number of offspring. After deduplication and competition, the next generation of structures is produced. This cycle continues until a global minimum is attained under the specified convergence limit. The structure optimizations at this stage were performed using a relatively coarse DFT method. Specifically, the B3LYP hybrid functional [80,81] with the LANL2DZ basis set [82] for Au and the 6–31+G* basis set [83,84,85] for O were utilized. For each Au_1–5_O*_x_*^−/+/0^ (*x* = 1–2), the program explored structure candidates in the two lowest-lying spin multiplicities, and for each Au_1–5_O*_x_*^−/+/0^ (*x* = 3–4), the program explored structure candidates in the three lowest-lying spin multiplicities. When conducting a structural search for the system containing three to four O atoms, the randomly generated structures consist of either all the O atoms being randomly dispersed or two of the O atoms combined as an O_2_ unit being adsorbed on the remaining gold oxide clusters containing a single O atom or two O atoms.
(4) All structures that were relatively stable (within approximately 1.0 eV of the lowest-lying one) underwent further optimization and scrutiny at a more sophisticated theory level, in which the B3LYP hybrid functional in combination with the def2-SVP basis set for Au and the def2-TZVP basis sets for O [86,87] was utilized. Scalar and spin-orbital relativistic effects of Au were addressed through energy consistent relativistic pseudopotentials. The ultimate global minima were validated via vibrational mode analysis, confirming the absence of imaginary frequencies.
(5) The adsorption energies of O_2_ on specific structures were calculated based on the Hartree–Fock energies corrected by the zero-point energies from frequency analyses. The formula for calculating the adsorption energy is the sum of the energies of the gold oxide cluster and O_2_, minus the energy of the compound after adsorption. The distribution of charges localized on the adsorbed O_2_ and the Au atom of the -O-Au sites were examined using the Natural Bond Analysis method [88]. The density of state (DOS) spectrum was obtained by broadening the calculated Kohn–Sham (KS) orbitals from the more sophisticated theory level using the Gaussian function with a FWHM of 0.1 eV. The position of HOMO in the DOS spectrum has been corrected using the clusters’ vertical detachment energy (VDE) values. All DFT calculations were performed using the Gaussian 09 program [89], and the DOS spectra were generated from the calculation results using the Multiwfn software [90].

## 4. Conclusions

Using an improved genetic algorithm program combined with DFT methods, we conducted extensive calculations on the structures of Au_1–5_O_1,2_^−/+/0^ and their corresponding products after adsorbing an O_2_, Au_1–5_O_3,4_^−/+/0^. The preferred adsorption sites and the charge-dependence of the adsorption strengths and the activation degrees were analyzed. The conclusions are as follow:

1. Regardless of the charge states of gold oxide clusters, the -O-Au sites are inevitably the primary sites for O_2_ adsorption.
2. The charge states of gold oxide clusters determine the bonding strengths and the activation degrees of the adsorbed O_2_. For anionic gold oxide clusters, the occurrence of electron transfer from the -O-Au sites to the adsorbed O_2_ leads to the formation of typical chemical bonds and high activation degrees of O_2_. For both cationic and neutral gold oxide clusters, their interactions with O_2_ are predominantly electrostatic. More positive charges on the Au atom of -O-Au sites in the cationic clusters lead to stronger binding energies than those of corresponding neutral ones. Meanwhile, the lower electron densities around the Au atom of -O-Au sites in the cationic clusters make electron transfer to O_2_ more unlikely, and O_2_ activation on the cationic gold oxide clusters is less effective than those in neutral species.

These findings could deepen the understanding of intricate charge effects on the ability of active sites on gold-based catalysts to activate O_2_ and offer pivotal information to reveal their catalytic mechanisms at the atomic and molecular level.

## Figures and Tables

**Table 1:** The adsorption energies (E*_a_*), the bond lengths (BL_O-O_), the NPA charges (Charge_O-O_), and the spins (Spin_O-O_) of the O_2_ units adsorbed on the -O-Au sites of the Au*_n_*O^−/+/0^ shown in Figure 1, Figure 2 and Figure 3.

Au*_n_*O^−/+/0^ + O_2_ Corresponding Pro		E*_a_* (eV)	BL_O-O_ (Å)	Charge_O-O_ (a.u.)	Spin_O-O_ (a.u.) (a.u.)
Anions	1-3-G-T	1.45	1.329	−0.720	0.986
	3-3-G-T	0.77	1.321	−0.626	1.038
					
Cations	1-3-G-Quint	0.78	1.207	+0.129	1.937
	2-3-G-D	0.64	1.207	+0.101	1.984
	3-3-G-T	0.56	1.211	+0.080	1.888
	4-3-G-Q	0.53	1.210	+0.076	1.901
	5-3-G-T	0.51	1.212	+0.061	1.928
	1-4-G-Quint	0.60	1.207	+0.096	1.896
					
Neutrals	1-3-G-Q	0.39	1.225	−0.065	1.670
	2-3-G-T	0.38	1.225	−0.071	1.729
	4-3-a-T	0.36	1.226	−0.099	1.732

## Data Availability

Data are contained within the article and Supplementary Materials.

## Supplementary Materials

The following supporting information can be downloaded at: https://www.mdpi.com/article/10.3390/molecules29071645/s1, Figure S1: Low-lying structures of Au_1-2_O*_x_*^−^ (*x* = 1–4). Figure S2: Low-lying structures of Au_3_O*_x_*^−^ (*x* = 1–4). Figure S3: Low-lying structures of Au_4_O*_x_*^−^ (*x* = 1–4). Figure S4: Low-lying structures of Au_5_O*_x_*^−^ (*x* = 1–4). Figure S5: Low-lying structures of Au_1-2_O*_x_*^+^ (*x* = 1–4). Figure S6: Low-lying structures of Au_3_O*_x_*^+^ (*x* = 1–4). Figure S7: Low-lying structures of Au_4_O*_x_*^+^ (*x* = 1–4). Figure S8: Low-lying structures of Au_5_O*_x_*^+^ (*x* = 1–4). Figure S9: Low-lying structures of Au_1-2_O*_x_* (*x* = 1–4). Figure S10: Low-lying structures of Au_3_O*_x_* (*x* = 1–4). Figure S11: Low-lying structures of Au_4_O*_x_* (*x* = 1–4). Figure S12: Low-lying structures of Au_5_O*_x_* (*x* = 1–4).
