# Supplementary material for: Adsorption of O2 on the Preferred -O-Au Sites of Small Gold Oxide Clusters: Charge-dependent Interaction and Activation
Source: Molecules. 2024 Apr 6;29(7):1645. doi: 10.3390/molecules29071645 (PMC11013888; doi:10.3390/molecules29071645)
Supplement: Supplementary file 1 [file molecules-29-01645-s001.zip › molecules-2936815-supplementary.pdf]

Adsorption of O_2 on the Preferred -O-Au Sites of Small Gold Oxide Clusters: Charge-dependent Interaction and Activation

Lulu Huang, Wen Liu, Xiaopeng Xing[*]

Shanghai Key Lab of Chemical Assessment and Sustainability, Department of Chemistry, Tongji University, 1239 Siping Road, Shanghai, 200092 (P. R. China).

[*]Authors to whom correspondence should be addressed. Tel.: +86-021-65981097;

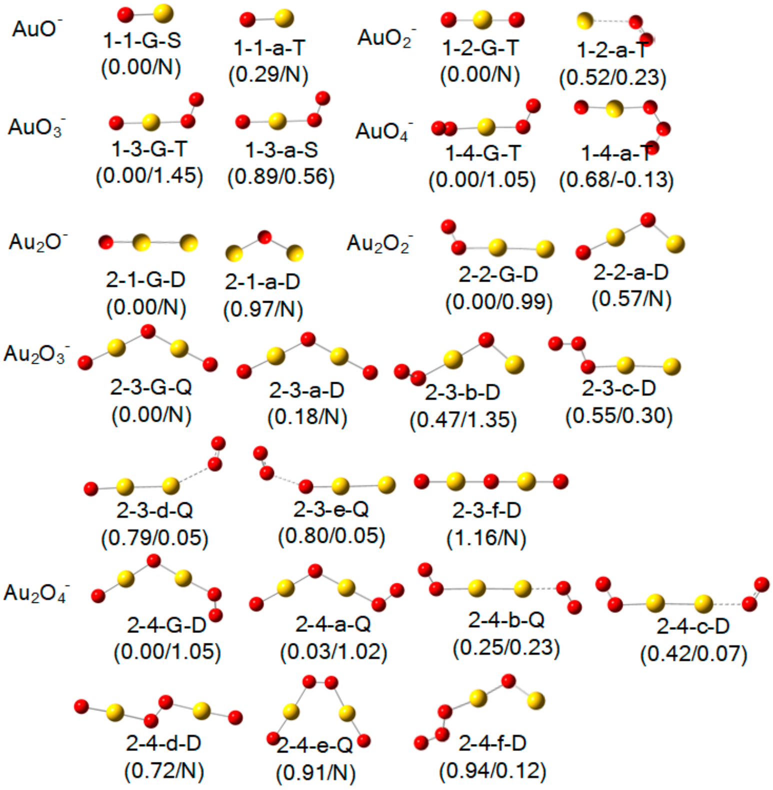

Figure S1. Low-lying structures of $Au_{1\text{-}2}O_x^-$ (x = 1-4) according to calculations at the B3LYP level with the basis sets of def2-SVP for Au, and def2-TZVP for O. In the labels of the structures, the first two numerals indicate the number of the gold atoms and that of the oxygen atoms, respectively; the third part "G/a/b..." means that this structure is the lowest-lying, the second lowest-lying, or the third lowest-lying one among all structural candidates; the fourth part indicates the spin-multiplicity, in which "S", "D", "T", "Q", and "Quint" stand for singlet, doublet, triplet, quartet, and quintet, respectively. The two numerals in the parenthesis of each label indicate the energy (in eV) relative to the lowest-lying structure and the adsorption energy (in eV) of O_2 (if there is such a unit and the left part didn't dramatically change when the O_2 is removed), respectively.

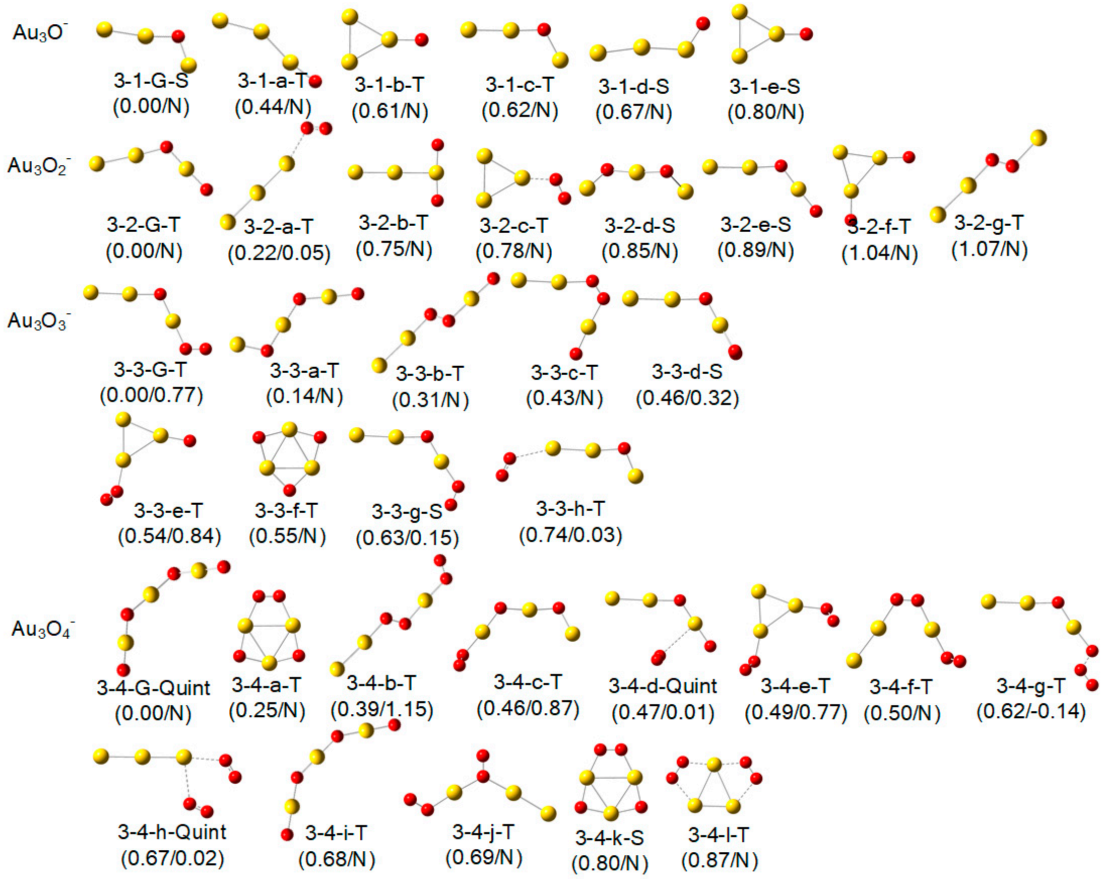

Figure S2. The structure with the low-lying energy of $Au_3O_x^-$ (x = 1-4). The meanings of the symbols, letters and numbers used in Figure S2 are consistent with those in Figure S1. See Figure S1 for details.

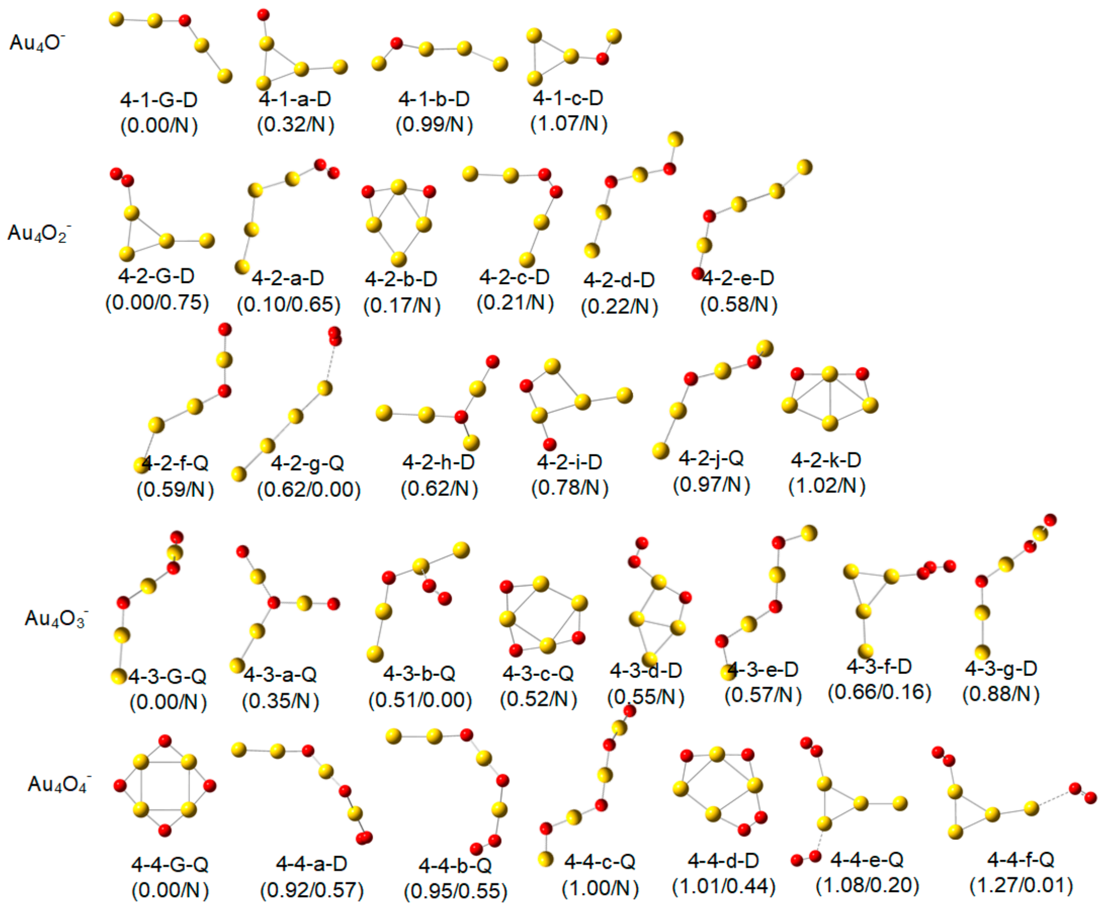

Figure S3. The structure with the low-lying energy of $Au_4O_x^-$ (x = 1-4). The meanings of the symbols, letters and numbers used in Figure S3 are consistent with those in Figure S1. See Figure S1 for details.

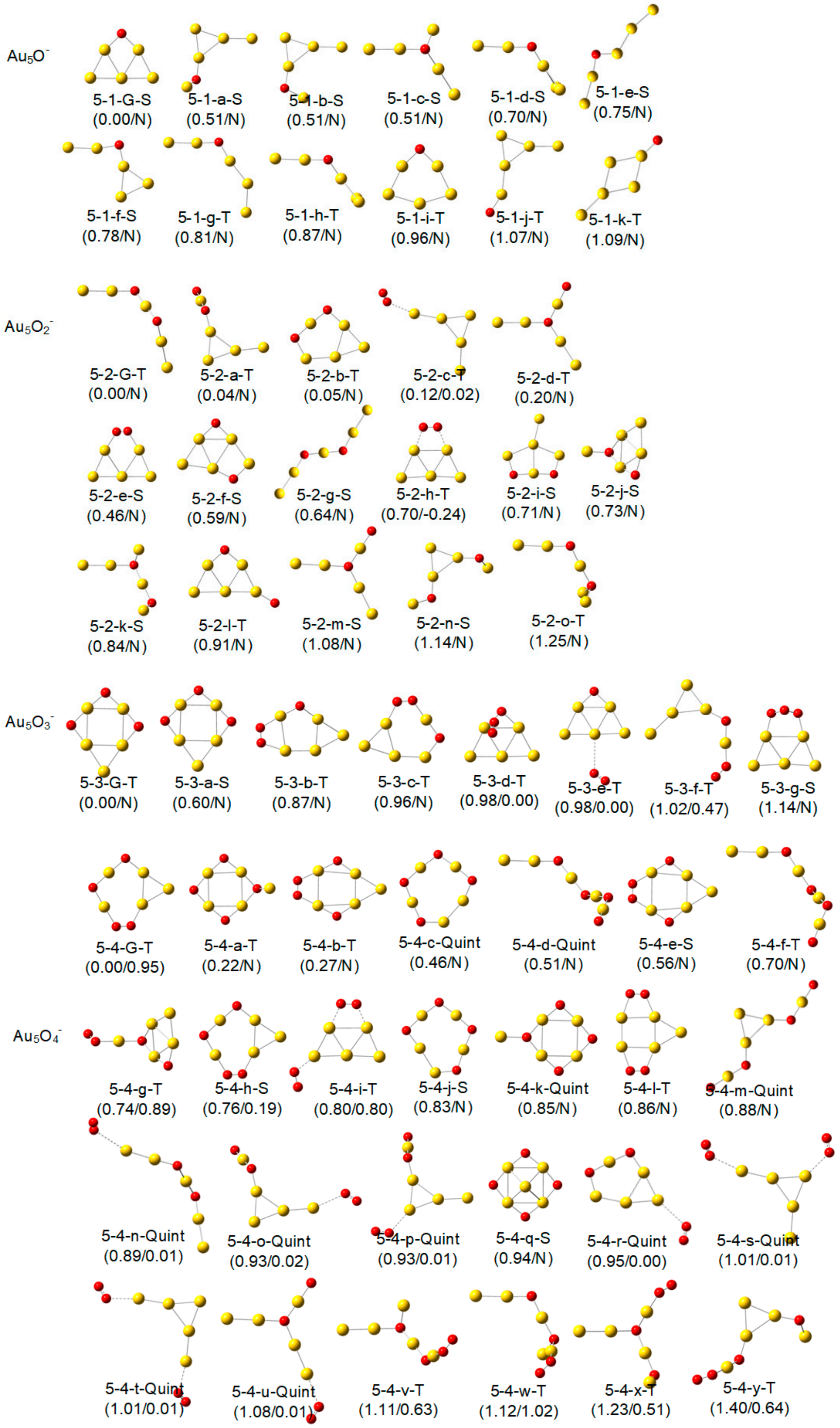

Figure S4. The structure with the low-lying energy of $Au_5O_x^-$ (x = 1-4). The meanings of the symbols, letters and numbers used in Figure S4 are consistent with those in Figure S1. See Figure S1 for details.

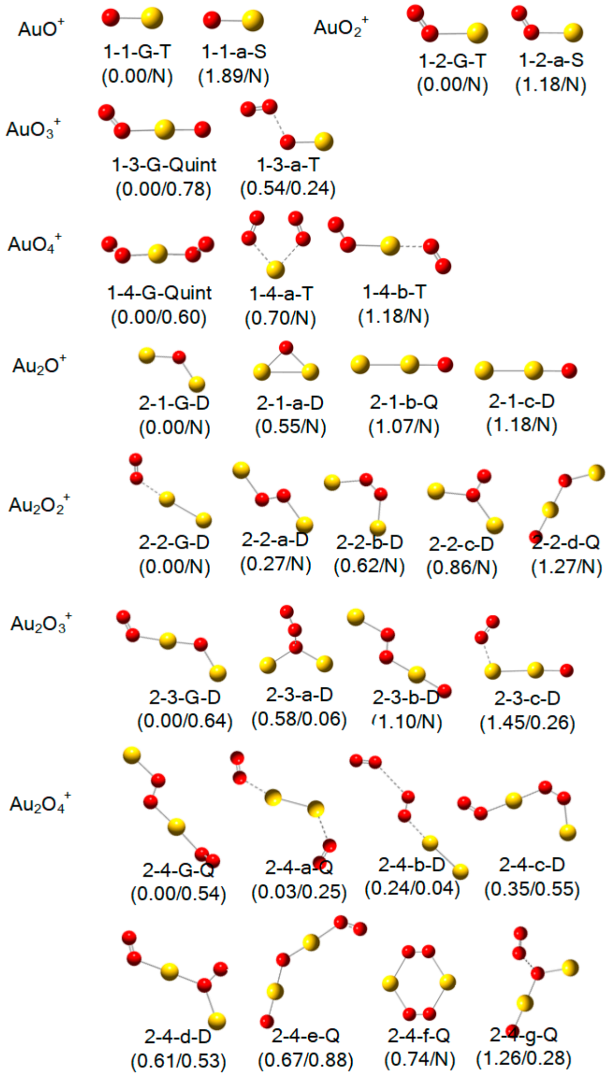

Figure S5. The structure with the low-lying energy of $Au_{1\text{-}2}O_x^+$ (x = 1-4). The meanings of the symbols, letters and numbers used in Figure S5 are consistent with those in Figure S1. See Figure S1 for details.

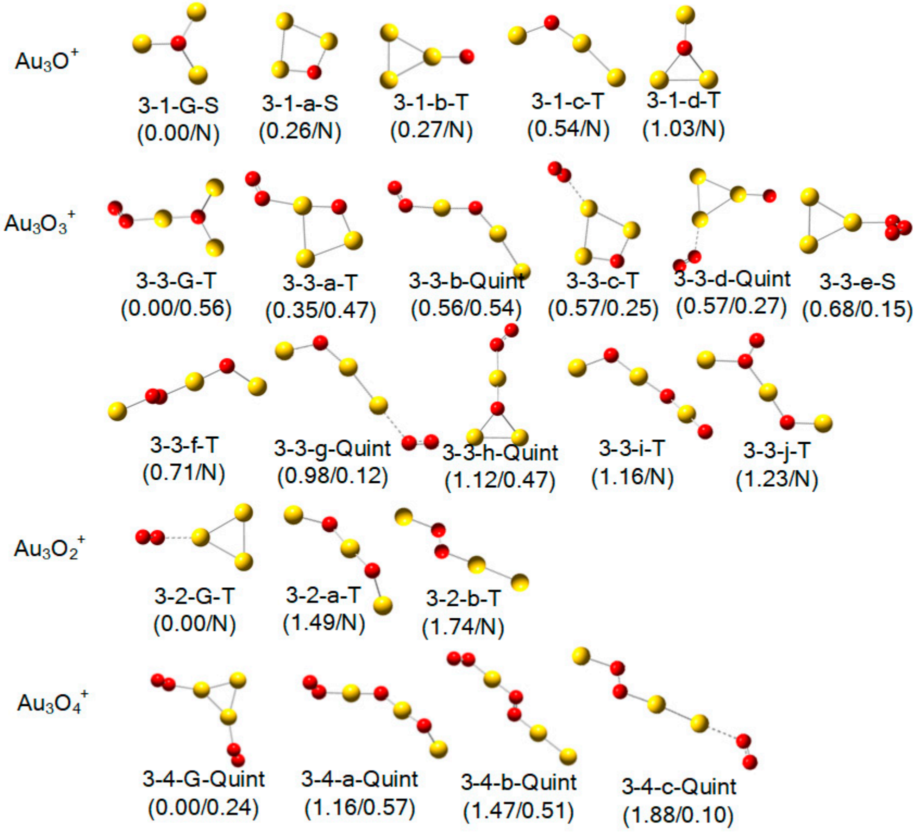

Figure S6. The structure with the low-lying energy of $Au_3O_x^+$ (x = 1-4). The meanings of the symbols, letters and numbers used in Figure S6 are consistent with those in Figure S1. See Figure S1 for details.

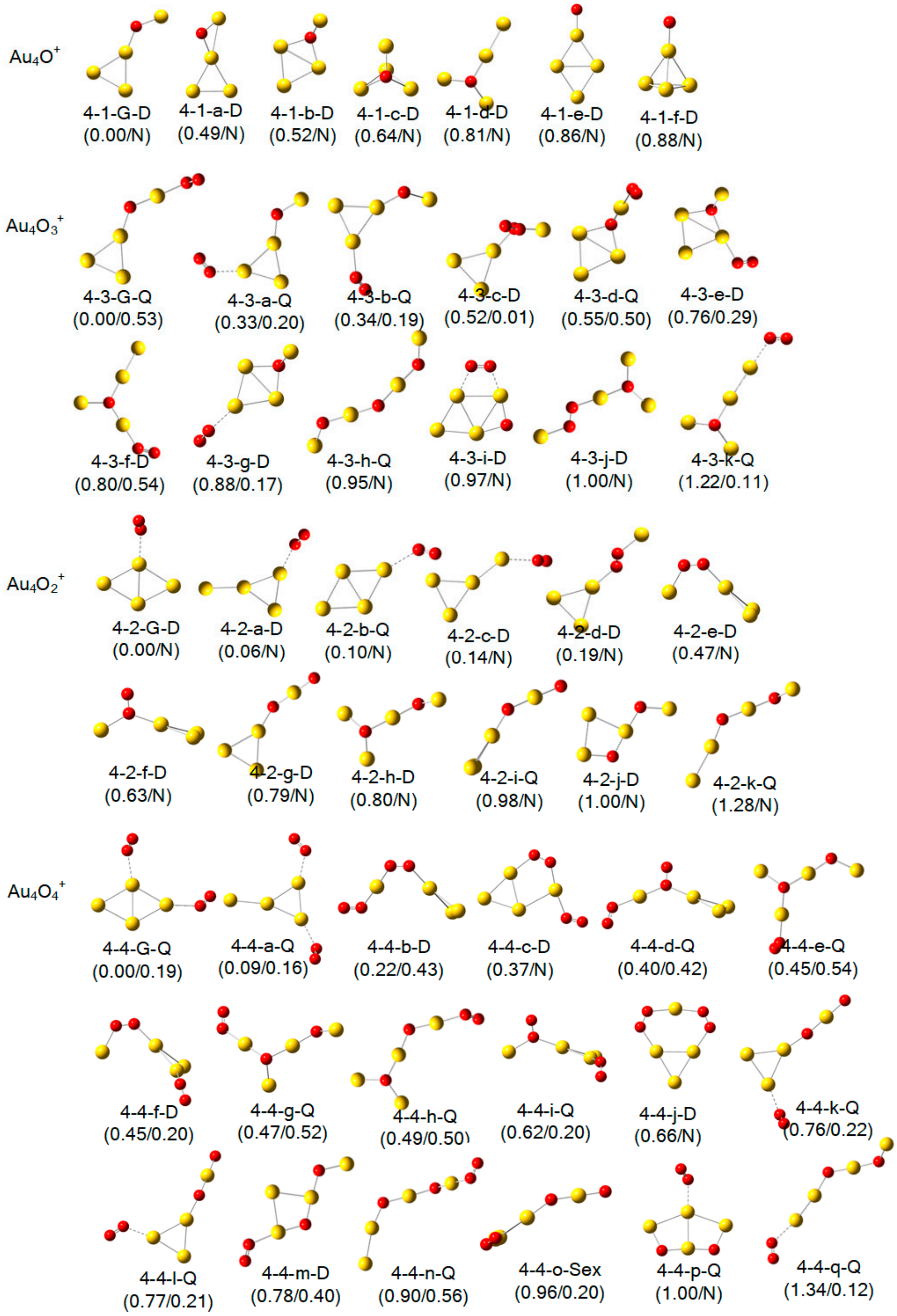

Figure S7. The structure with the low-lying energy of $Au_4O_x^+$ (x = 1-4). The meanings of the symbols, letters and numbers used in Figure S7 are consistent with those in Figure S1. See Figure S1 for details.

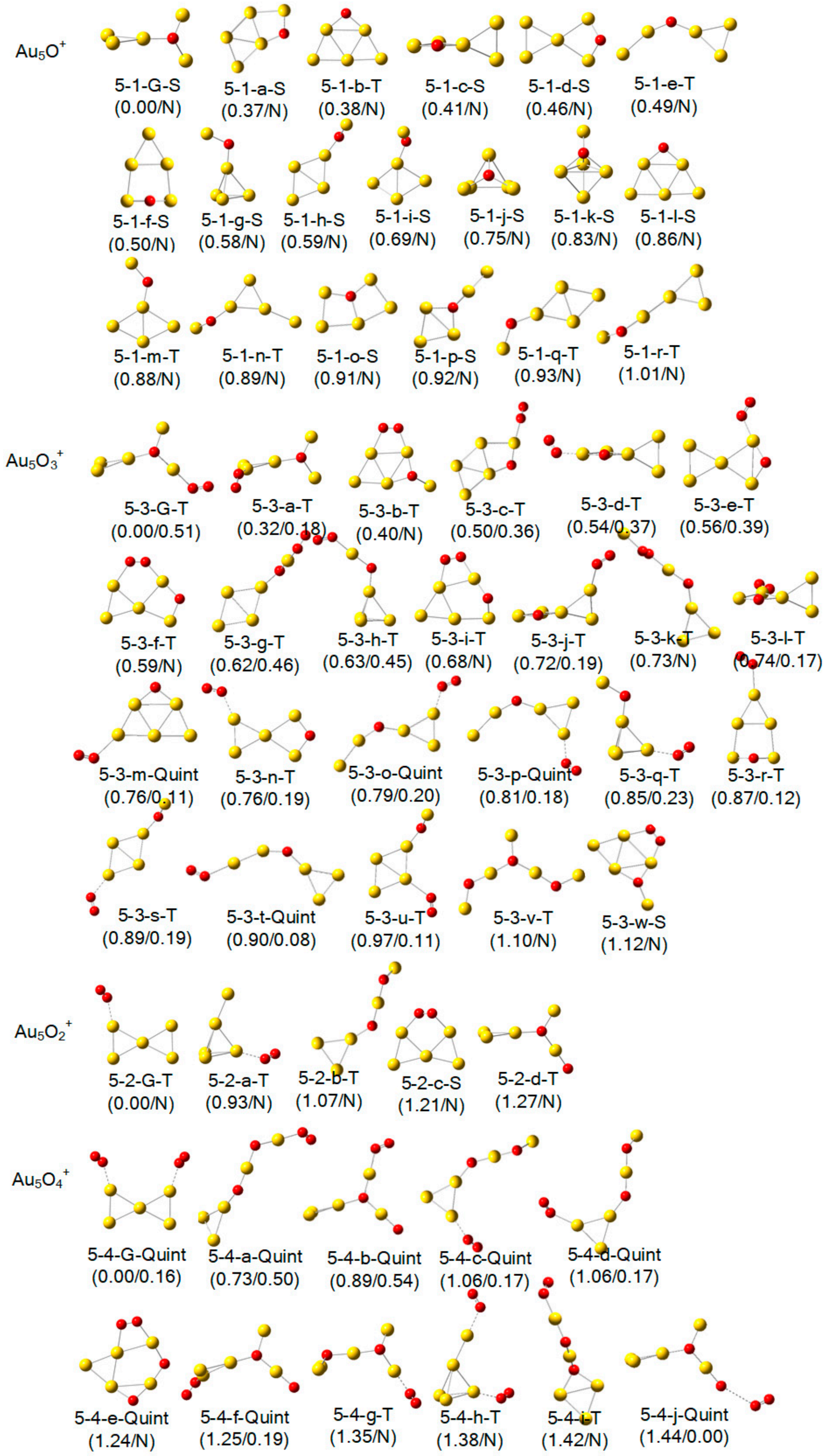

Figure S8. The structure with the low-lying energy of $Au_5O_x^+$ (x = 1-4). The meanings of the symbols, letters and numbers used in Figure S8 are consistent with those in Figure S1. See Figure S1 for details.

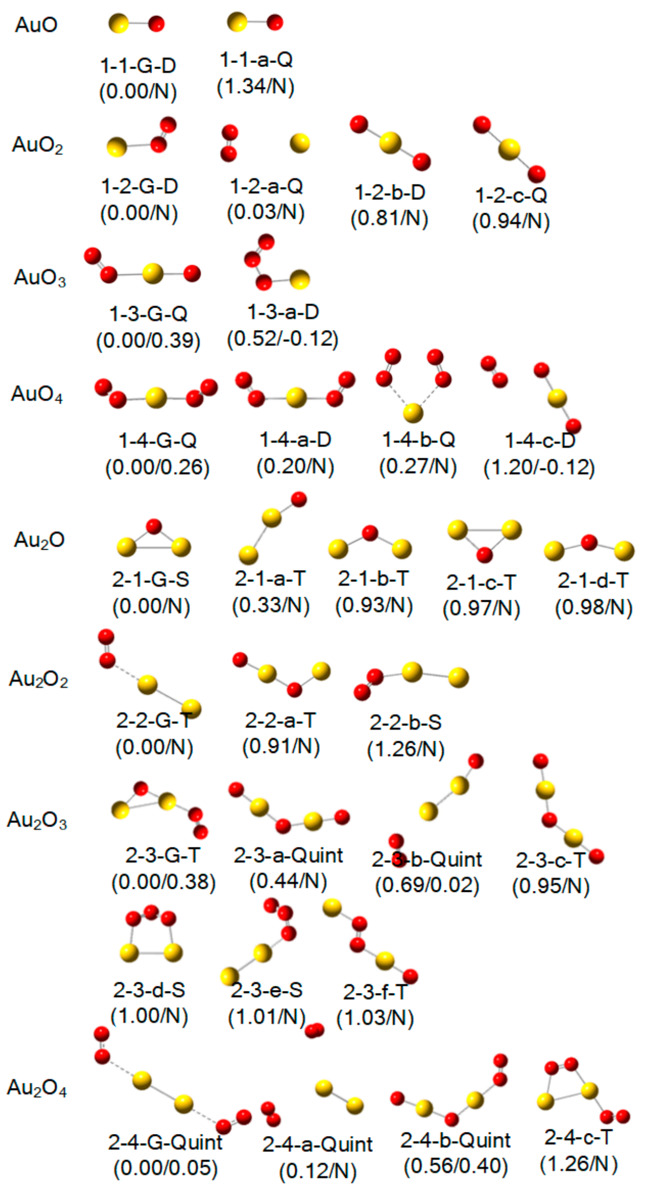

Figure S9. The structure with the low-lying energy of $Au_{1\text{-}2}O_x$ (x = 1-4). The meanings of the symbols, letters and numbers used in Figure S9 are consistent with those in Figure S1. See Figure S1 for details.

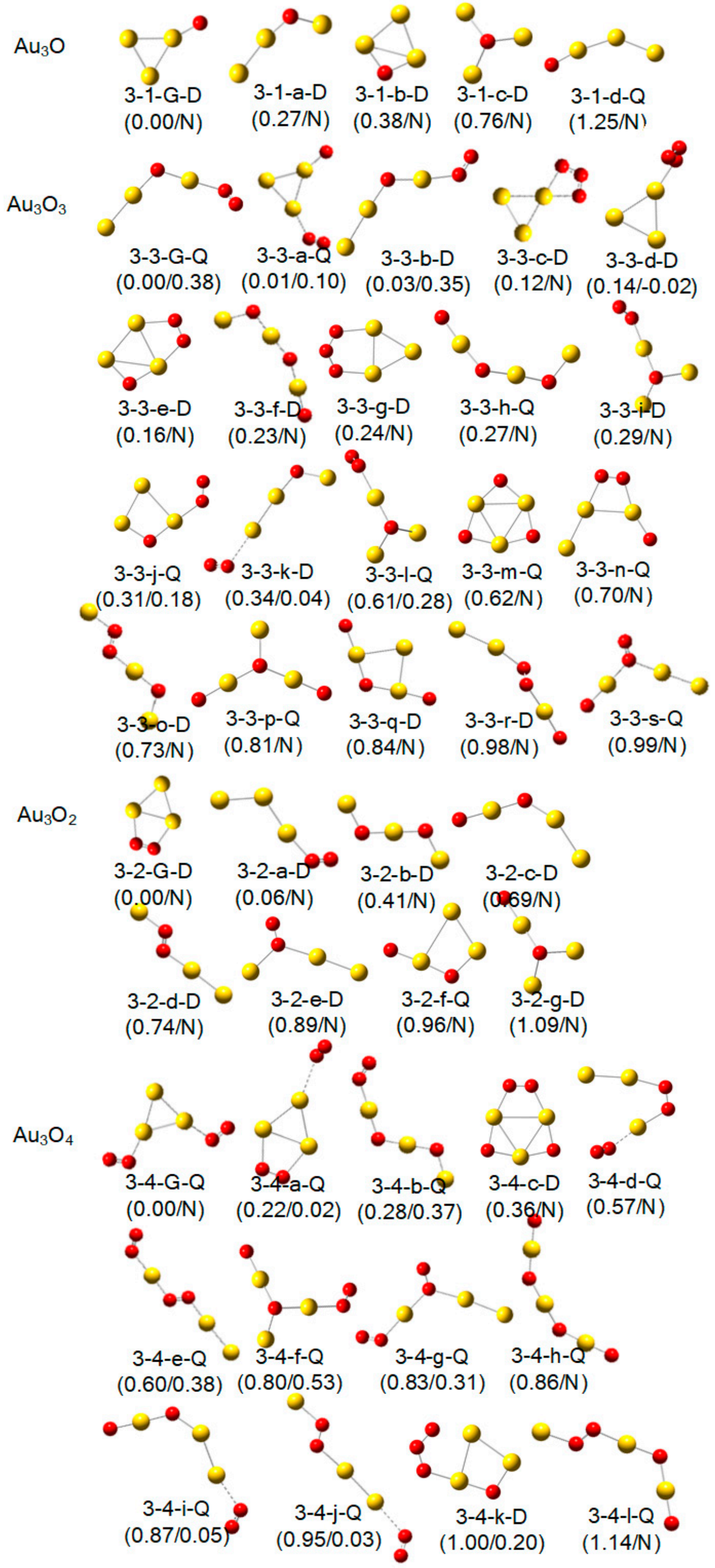

Figure S10. The structure with the low-lying energy of Au_3O_x (x = 1-4). The meanings of the symbols, letters and numbers used in Figure S10 are consistent with those in Figure S1. See Figure S1 for details.

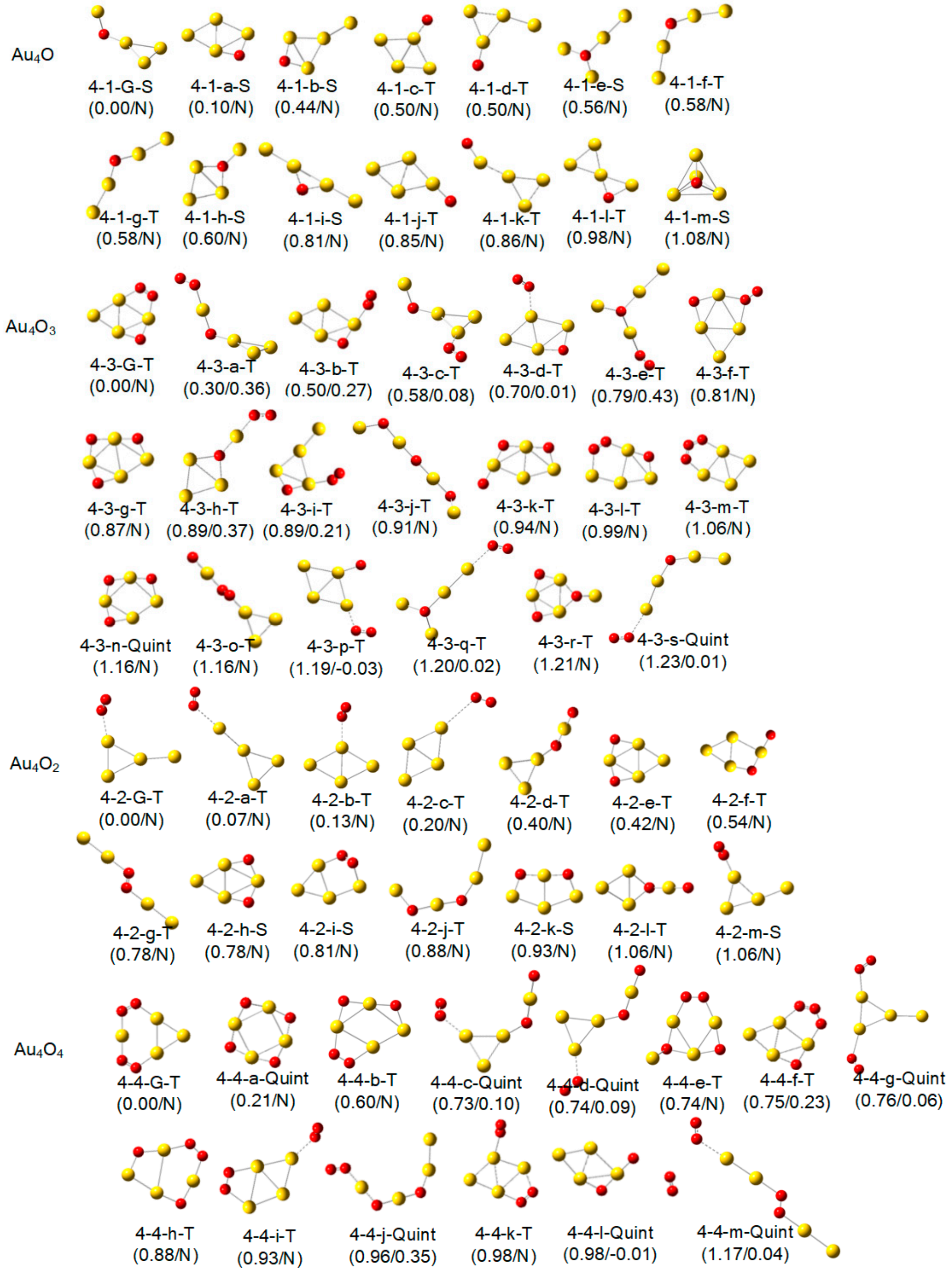

Figure S11. The structure with the low-lying energy of Au$_4$O$_x$ (x = 1-4). The meanings of the symbols, letters and numbers used in Figure S11 are consistent with those in Figure S1. See Figure S1 for details.

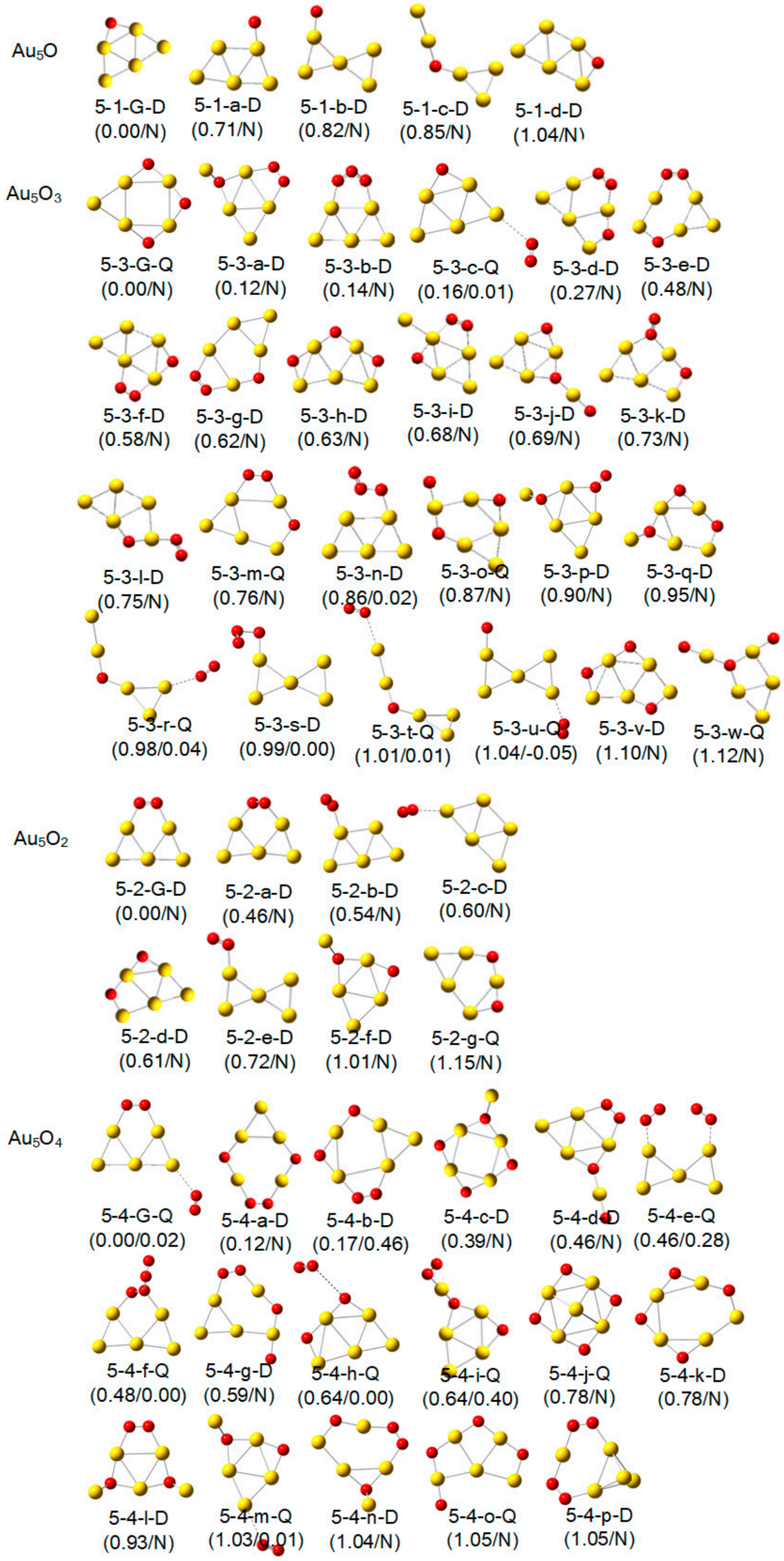

Figure S12. The structure with the low-lying energy of Au_5O_x (x = 1-4). The meanings of the symbols, letters and numbers used in Figure S12 are consistent with those in Figure S1. See Figure S1 for details.
